# Assessing structural and reported biosecurity measures in Irish broiler farms from 2019 to 2023

**DOI:** 10.1186/s13620-025-00318-y

**Published:** 2025-12-10

**Authors:** Lianjie Wei, Edgar Garcia Manzanilla, Alberto Allepuz Palau, Carla Correia-Gomes

**Affiliations:** 1 Pig Development Department, Teagasc, Moorepark, Fermoy, Co. Cork P61C996 Ireland; 2 School of Veterinary Medicine, University College Dublin, Belfield, Dublin 4 D04 W6F6 Ireland; 3 Departament de Sanitat I Anatomia Animals, Facultat de Veterinària, Universitat Autònoma de Barcelona, Cerdanyola del Vallès, Barcelona, 08193 Spain; 4 Animal Health Ireland, 2-5 Archways, Carrick On Shannon, Ireland

**Keywords:** Biocheck.Ugent, Biosecurity, Broilers, Ireland, Poultry

## Abstract

**Background:**

Biosecurity measures are essential to prevent the introduction and spread of pathogens within (internal biosecurity) and between (external biosecurity) broiler farms. Implementing effective biosecurity practices not only protects animal health but also enhances productivity, welfare, and farm sustainability in general. This study assesses the temporal trends in biosecurity scores in Irish broiler farms from 2019 to 2023 using the Biocheck.UGent tool and identifies areas for improvement. The analysis includes data from 403 broiler farms, as well as recommendations provided by private veterinary practitioners (PVPs) to enhance biosecurity.

**Results:**

The results show an overall upward trend in biosecurity scores over the study period. Internal biosecurity scores were consistently higher than external scores. Median internal scores increased from 60 (over 100) in 2019 to 75 in 2023 (*P* < 0.05). External scores increased from 50 to 65 in the same period (*P* < 0.05). Farms that underwent at least three assessments showed increases in median total scores of roughly 10 points after the first visit (*P* < 0.05). However, certain biosecurity categories, particularly cleaning and disinfection with medians over years remaining below 70, received consistently low scores despite frequent recommendations for improvement by the PVPs.

**Conclusions:**

The findings suggest that, while progress has been made, further efforts are needed to enhance biosecurity practices, particularly in areas with persistent low scores, such as depopulation of broilers and cleaning and disinfection. PVPs should provide more targeted recommendations for these categories and support farmers in effectively implementing these practices.

## Introduction

In Ireland, poultry farming is a vital component of the agricultural sector, making substantial contributions to the national economy [10]. Irish poultry production is vertically integrated, with integrator companies coordinating chick supply, feed delivery, veterinary support, catching and transport. In 2023, this sector displayed a robust growth trajectory, with export values escalating by approximately 7% to exceed 170 million and prices surging by 12% [13]. However, the growth of the sector is restrained by infectious diseases such as highly pathogenic avian influenza (HPAI), infectious bronchitis (IB) and infectious laryngotracheitis (ILT), as well as bacterial pathogens including Salmonella and Campylobacter [16]. The poultry sector consists of broiler and layer systems, and this study focuses on broiler farms to assess biosecurity performance.

Biosecurity encompasses a suite of measures designed to sustain animal health and is recognised as an efficient strategy to control infectious diseases [1, 28]. Biosecurity is usually divided in external biosecurity and internal biosecurity. External biosecurity focuses on preventing the introduction of pathogens from external sources, covering aspects such as the entry of vehicles and visitors, farm location, and supply of materials. Internal biosecurity aims to curb the spread of pathogens within the farm or herd, emphasizing procedures like sanitation, disinfection, and husbandry [3, 28]. In recent years, broader frameworks have been proposed for livestock biosecurity. For example, Saegerman et al. [26] have extended the focus beyond preventing pathogen introduction and spread to animals to also addressing zoonotic hazards and environmental contamination.

Implementing biosecurity measures can confer further positive effects. Researchers from several countries have revealed that implementation of biosecurity measures mitigates the risk of infectious disease occurrence and transmission [17, 18, 21]. Moreover, adherence to biosecurity measures has been linked to reduced antimicrobial usage (AMU), thus helping to reduce the risk of antimicrobial resistance (AMR) [2, 14, 19]. Robust biosecurity practices also improve farm efficiency and productive performance, thereby benefiting profitability [20, 29].

Questionnaires or checklists can be used to enquire about compliance with biosecurity protocols. These assessments, whether quantitative or qualitative, are based on information provided by farmers or advisors regarding the implementation of biosecurity practices. From these evaluations, areas of weakness may be identified [1, 25]. Consequently, farm advisors can provide targeted recommendations, and regulatory bodies may implement training and, if necessary, impose penalties for farmers and workers to improve their performance [9].

Building on the National Farmed Animal Biosecurity Strategy (2021–2024) [4], Ireland implemented a programme to routinely assess poultry-farm biosecurity with the Biocheck.UGent tool (https://biocheckgent.com/en). Under the Targeted Advisory Service on Animal Health (TASAH), commercial broiler farms are eligible for one funded assessment per calendar year. This framework provides a consistent dataset that underpins the present study. The primary goals of this study are to describe the evolution of Biocheck.UGent survey scores in Irish broiler farms from 2019 to 2023, summarise the types of recommendations provided, identify areas of weakness in structural and reported biosecurity measures and highlight areas that have improved over time.

## Materials and methods

### Type of system used to assess biosecurity

The Biocheck.UGent scoring system [12, 27] was employed to assess biosecurity in broiler farms. This risk-based assessment tool encompasses a broad spectrum of questions related to biosecurity practices, covering eight external and three internal categories specific to broilers. Scores assigned based on advisor responses range from 0 to 100, indicating the lowest and highest levels of biosecurity compliance, respectively. Weighting factors are subsequently applied to obtain comprehensive scores for external, internal, and overall biosecurity for each farm (see more details of the system at https://biocheckgent.com/en).

### Implementation of the assessments

Assessments are conducted by trained private veterinary practitioners (PVPs) supported by TASAH under the Rural Development Programme. Assessments are conducted in person, with one visit per eligible broiler farm per calendar year. This initiative is coordinated by Animal Health Ireland (AHI), which delivers two training sessions on average to PVPs and ensures that payments are made directly to the them upon the completion of their assessments. Funding is provided exclusively to PVPs who have undergone specific training for these evaluations. Farm owners interested in these services can consult a directory of qualified PVPs on the AHI website (https://animalhealthireland.ie/programmes/ibr/ibr-tasah-trained-veterinary-practitioners/) and can engage their services directly. During each visit, PVPs tour the site and complete the Biocheck.UGent questionnaire with the farm owner or manager, and completion requires approximately 90 min. These assessments are funded annually by the TASAH, ensuring regular reviews of biosecurity practices.

The assessment on farm consists in completing the Biocheck.UGent questionnaire and providing a maximum of three recommendations, agreed with the farmer, for improving biosecurity. PVPs organise the visits with the farm owner/manager, complete the assessment on farm, and input the questionnaire answers and agreed recommendations into the AHI web portal. The farmer then receives a report with the scores for his/her farm for the several biosecurity categories, including benchmarking of the farm results with the national average, and the recommendations agreed.

### Data collection and analysis

The data used in this study was extracted from the AHI database, which contains records of biosecurity assessments conducted on broiler farms in Ireland from 2019 to 2023. It should be noted that each year the number of farms surveyed varied, therefore there were numerous farms inspected multiple times.

The data was analysed for trends over time in two ways: comparing the scores between years including all farms available and then comparing the scores between visits including only those farms that had more than 2 visits. For this second cohort of farms, visits are not necessarily on consecutive years and only the last three assessments were considered in this analysis. But for the sake of simplicity these most recent three assessments were named as first, second and third visit, corresponding respectively to third most recent assessment, second most recent assessment and most recent assessment. To explore differences in biosecurity scores between years and visits, Kruskal–Wallis tests were performed, followed by Dunn’s post-hoc tests for pairwise comparisons where significant differences were detected. The analysis was carried out for external, internal, and overall biosecurity scores and then a similar approach was taken for each specific biosecurity category.

Recommendations made by PVPs were categorised, and bar plots were created to visualize the distribution of recommendation categories. Categorisation was conducted, manually, by the authors based on the categories given in the Biocheck.UGent questionnaires. In case a recommendation would not fit within the BiocheckUGent biosecurity categories it was categorised as ‘Others’. In case of doubt the categorisation was attributed by consensus between two of the authors (LW and CCG).

All analyses were performed in RStudio using packages ggplot2 [34], tidyverse [31], dplyr [33], patchwork [23], car [11], and dunn.test [8]. The alpha level used for determination of significance was 0.05.

## Results

### Farm characteristics

From 2019 to 2023, 403 broiler farms were assessed at least one time. Over the years, the number of farms assessed showed a generally increasing trend, starting at 49 in 2019, followed by 182 in 2020, 145 in 2021, 315 in 2022, and reaching 325 in 2023.

### Biosecurity scores by year

**Fig. 1:**
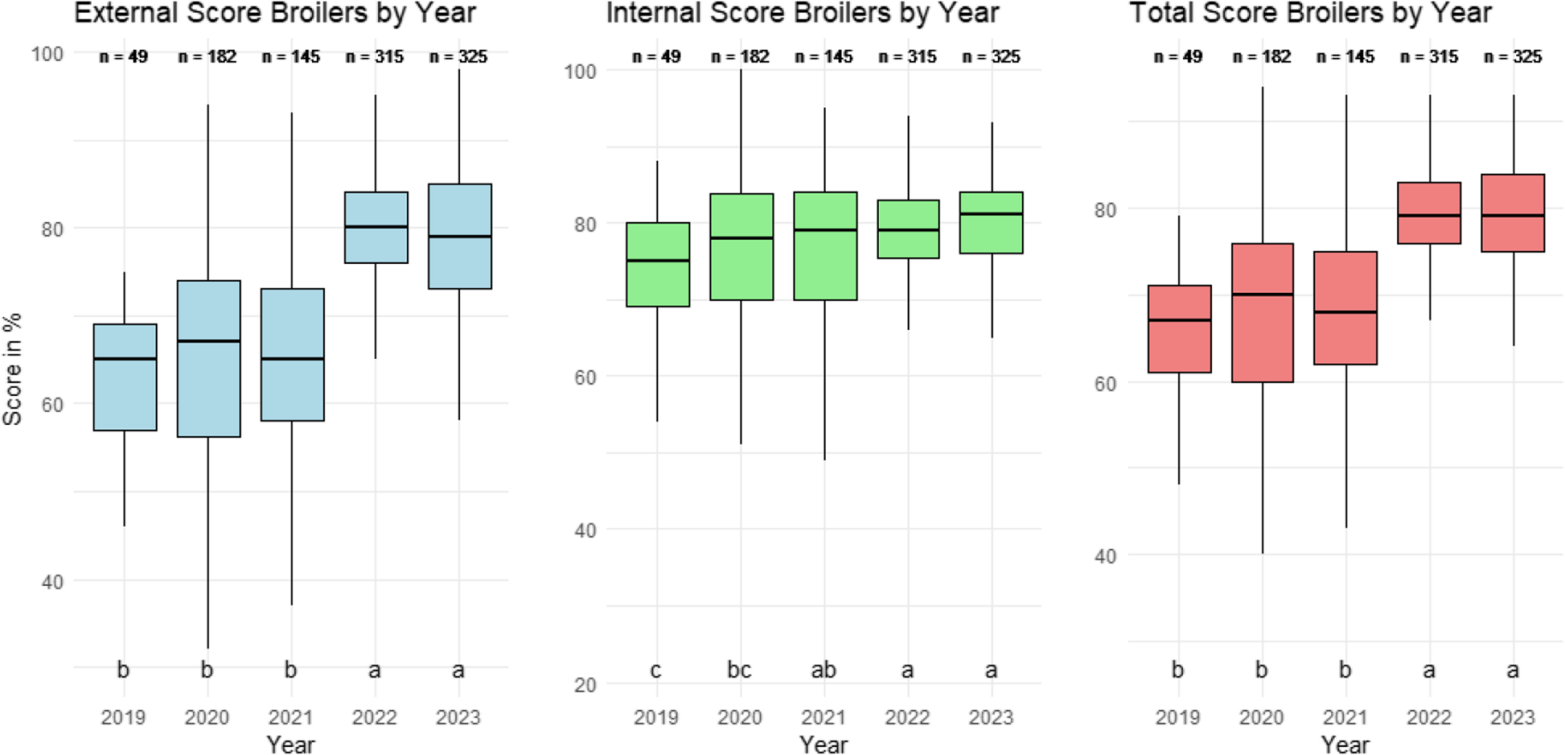
External (blue), internal (green) and total (red) biosecurity scores for broiler farms from 2019 to 2023. Numbers above the boxplot display the number of farms assessed per year. Letters (**a**, **b**, **c**) indicate differences. Groups with no common letters are significantly different (*p* < 0.05)

Figure 1 shows the temporal trends in external, internal and total biosecurity scores for broiler farms. An overall upward trend in medians in all biosecurity scores in broiler farms can be observed. There was an increase in external and total scores from 2021 to 2022, from approximately 65 to 80, with 2019, 2020, and 2021 showing lower scores than 2022 and 2023. By comparison, internal biosecurity scores progressed less but in a more regular basis (from 75 to 80) with 2019 scores being lower than 2021, 2022, and 2023 scores and 2020 scores being lower than 2022 and 2023.

**Fig. 2:**
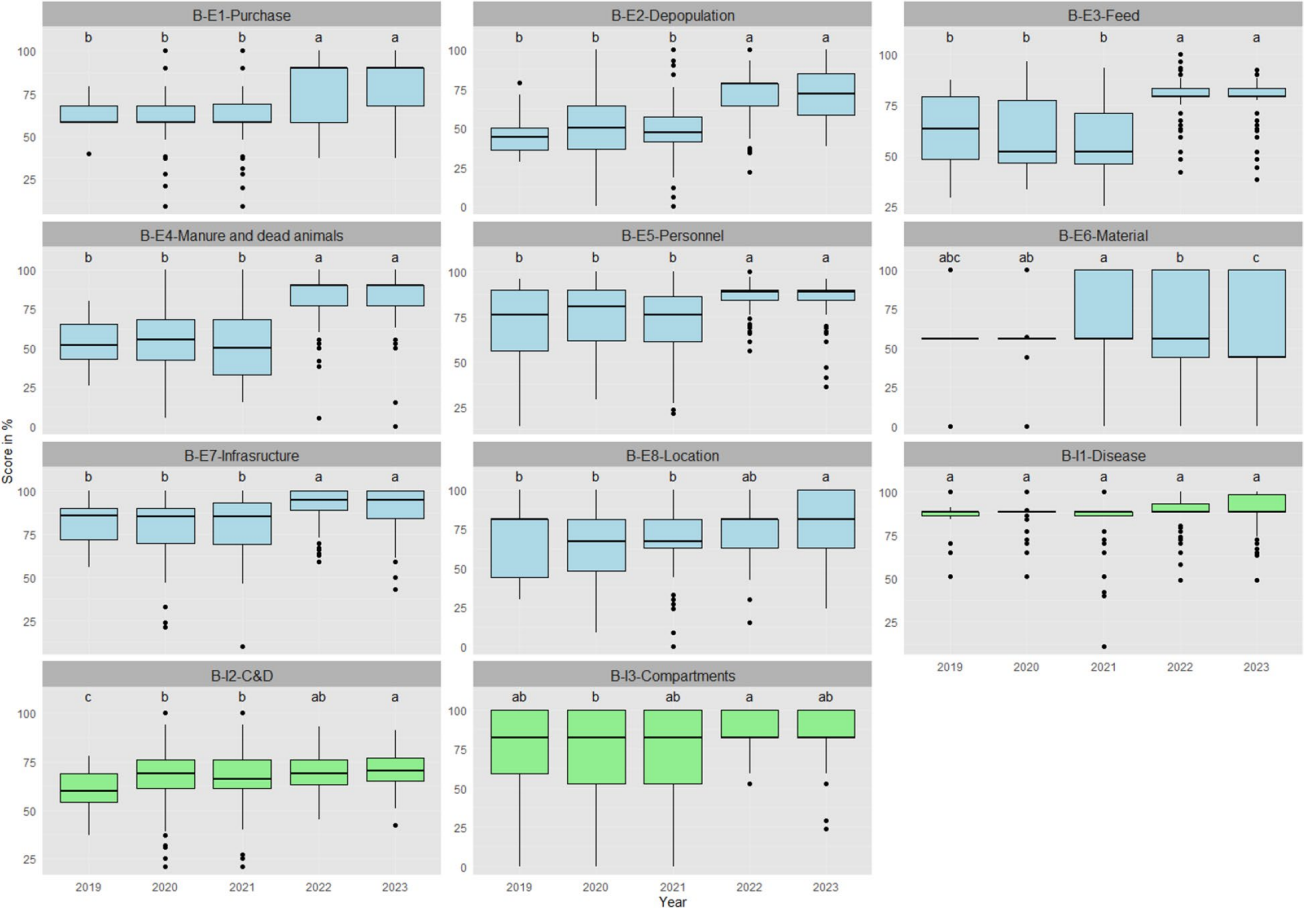
Distribution of the scores per category for external (blue) and internal (green) biosecurity for broiler farms from 2019 to 2023. Letters (**a**, **b**, **c**) indicate differences. Groups with no common letters are significantly different (*p* < 0.05). The full names of categories are: B-E1-Purchase of one-day-old chicks, B-E2-Depopulation of broilers, B-E3-Feed and water, B-E4-Removal of manure and dead animals, B-E5-Entrance of visitors and farm workers, B-E6-Material supply, B-E7-Infrastructure and biological vectors, B-E8-Location of the farm, B-I1-Disease management, B-I2-Cleaning and disinfection, B-I3-Materials and measures between compartments

Figure 2 shows the distribution of scores per biosecurity category from 2019 to 2023. When looking at the median value of specific biosecurity categories on broiler units, most external categories show the same trend as shown in Fig. 1, except for material supply (B-E6) and location of the farm (B-E8). Median scores of material supply remained low, with a drop in 2023 compared to 2020 to 2022. On the other hand, scores for the location of the farm in 2023 were higher than in previous years. Meanwhile, among categories that followed the general upwards trend, some had relatively low scores. For instance, scores for depopulation of broilers (B-E2) and supply of feed and water (B-E3) were lower than 60 from 2019 to 2021. For internal categories, there were no differences between years in scores for disease management (B-I1). Scores in 2022 were higher than in 2020 for materials and measures between compartments (B-I3). Cleaning and disinfection (B-I2) showed a slightly increasing pattern over the years, with 2020 and 2021 scoring higher than 2019, and 2022 and 2023 higher than 2020 and 2021, but they were all below 75.

### Trends over time per farm

**Fig. 3:**
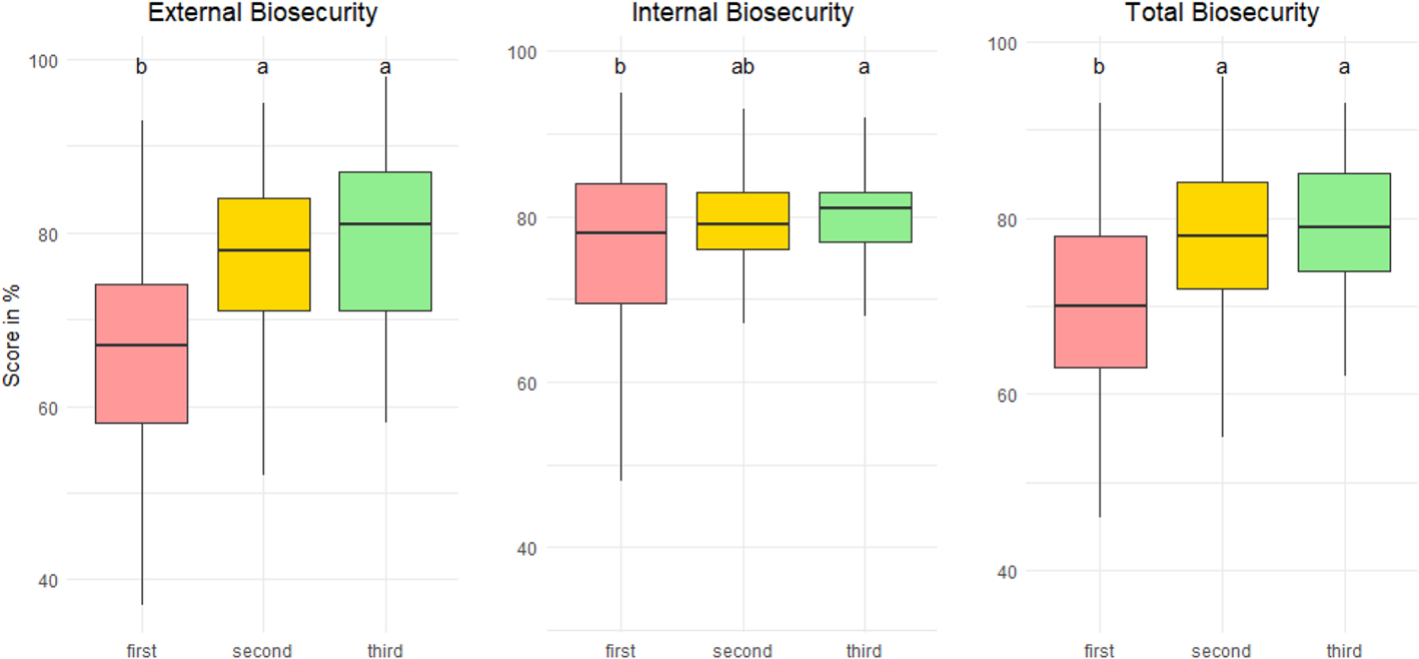
Biosecurity scores by the times of visits (last three most recent visits in sequential order from third most recent – first – to most recent – third) of 171 broiler farms that were visited at least three times. Letters (**a**, **b**, **c**) indicate differences. Groups with no common letters are significantly different (*p* < 0.05)

**Fig. 4:**
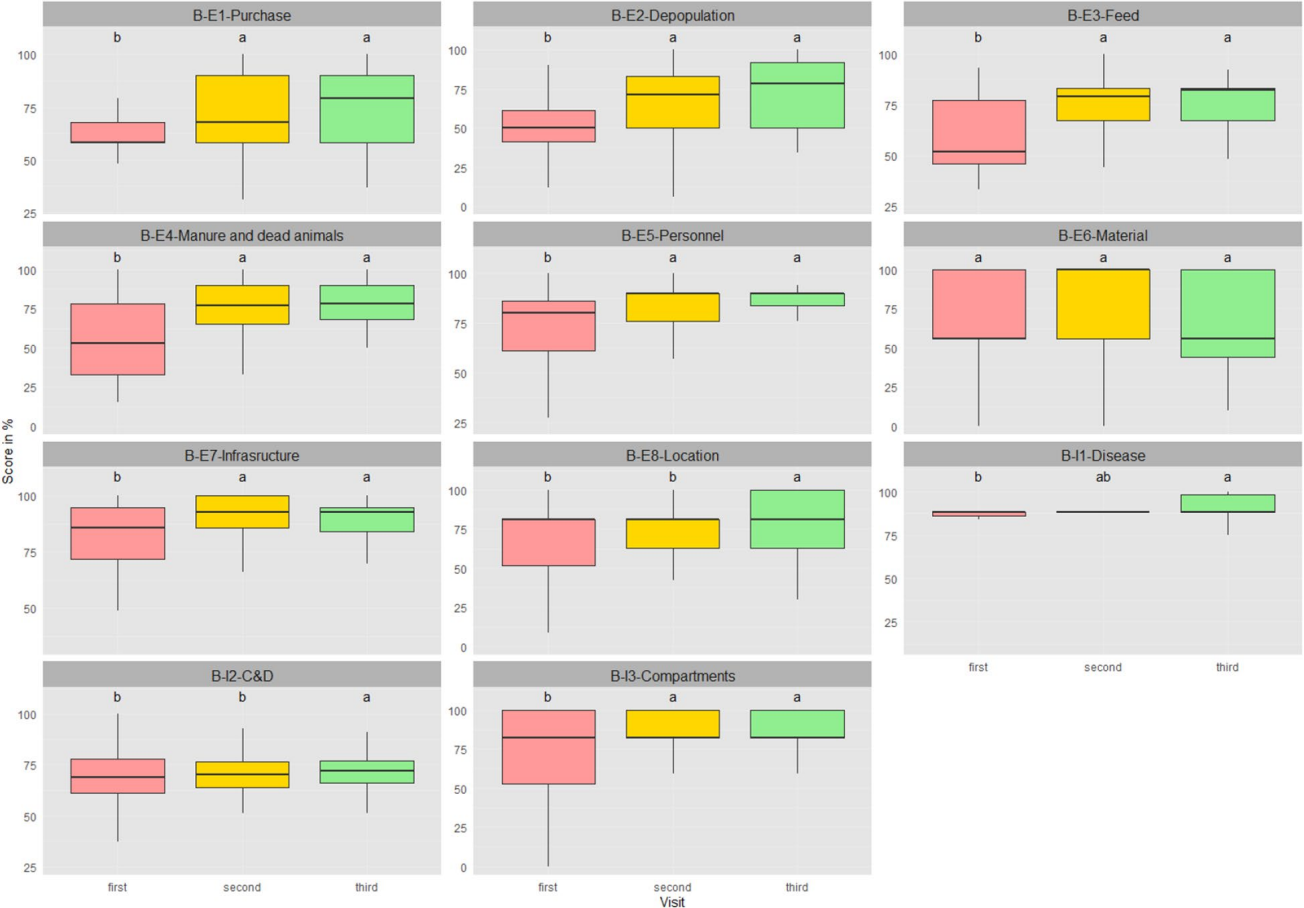
Distribution of the scores per category for external and internal biosecurity of 171 broiler farms that were visited at least three times (last three most recent visits in sequential order from third most recent – first – to most recent – third). Letters (**a**, **b**, **c**) indicate differences. Groups with no common letters are significantly different (*p* < 0.05). For full names of categories, see Fig. 2

One hundred and seventy-one broiler units have been assessed at least three times during the four-year period. The average time between the first and second visits was 447 days, while the average time between the second and third visits was 388 days. Figure 3 shows the results of biosecurity scores for the last three more recent visits for this cohort of 171 units. For external and total scores, improvements were observed between the first and second visits, while no differences were found between the second and third visits. For internal scores, no differences were found between the first and second visits, but the third visit showed higher scores than the first visit. No differences were observed between the second and third visits either. Figure 4 shows the distribution for each biosecurity category. Most of them illustrates the same trend as Fig. 3, but there are some exceptions. For instance, there was no difference in scores between visits for material supply (B-E6), and only scores on the third visit were higher than previous ones for the location of the farm (B-E8). Different trends were also observed in internal categories, with scores of the second visit lower than the third visit for cleaning and disinfection (B-I2), and higher than the first visit for materials and measures between compartments (B-I3).

### Distributions of recommendation categories

**Fig. 5:**
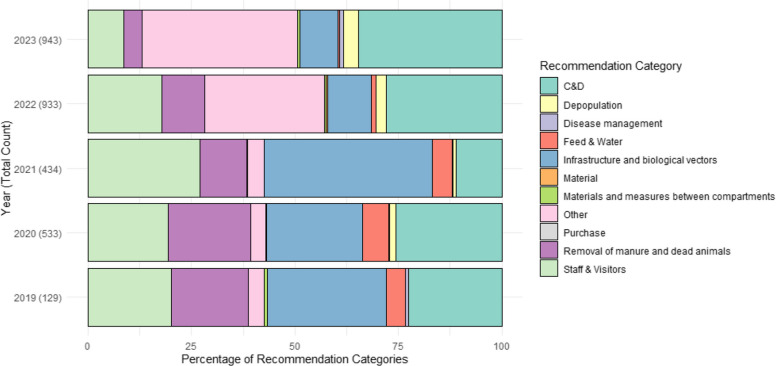
Distribution of the veterinary recommendations by category over the years

Figure 5 shows the distributions and total counts of recommendation categories for broiler farms by year. The most common recommendation category is cleaning and disinfection (C&D), which belongs to internal biosecurity. ‘Other’, including repairs for buildings and floors, takes the second position. The remaining distribution is predominantly related to external biosecurity, with major categories including infrastructure and biological vectors, and visitors and staff.

Based on the yearly distribution of the recommendations, infrastructure and biological vectors were the most frequent recommendations in 2019 (28.7%) and 2021 (40.6%) years. Cleaning and disinfection (C&D) was the highest category in 2020 (25.7%) and second highest in 2022 (28.0%) and 2023 (34.7%). In 2022 and 2023, 'Other' was the highest category (29.0% and 37.5%). Other categories with a high proportion of recommendations were staff and visitors, which was frequently recommended from 2019 to 2022 (always higher than 17.0%), and removal of manure and dead animals, which had figures close to 20% in 2019 and 2020.

## Discussion

In this study, biosecurity scores in commercial Irish broiler farms were assessed over time using a widely used biosecurity assessment system and areas for improvement were identified. These assessments began in 2019, with farms participating on a voluntary basis. Over the study period, 403 broiler farms underwent at least one biosecurity assessment, covering approximately 89.6% of commercial broiler farms in Ireland (based on Bord Bia 2022 figures – personal communication). The number of assessments generally increased over time, which likely reflects wider programme uptake, growing farmer engagement, improved PVP availability, and heightened risk awareness. However, there was a decrease in the numbers of farms doing the assessments in 2021. That year due to the outbreaks of high pathogenic avian influenza (HPAI) in the north of the country, PVPs were asked to suspend the biosecurity visits to avoid inadvertently contributing to the spread of the disease [24].

Internal biosecurity scored higher than external biosecurity for most of the years for broiler farms. However, it was noticeable the improvement in external biosecurity from 2021 to 2022, after the outbreaks of HPAI, when the industry was acutely aware that improvements were needed in external biosecurity to avoid or minimize further outbreaks after 2021 [5]. Additionally, Irish broiler production is vertically integrated, with integrators coordinating the supply chain and production activities. This structure allows greater governance over biosecurity practices and may help explain the marked improvement.

When comparing biosecurity scores per category, categories with weak compliance can be identified. For external biosecurity, it is noticeable that categories such as the purchase of one-day-old chicks, the entrance of visitors and farm workers, infrastructure and biological vectors boast high biosecurity scores. By contrast, practices regarding depopulation of broilers, supply of feed and water, and supply of materials indicate a need for further improvement. Some examples (based on questionnaire replies) of practices to improve are: ensuring that transport vehicles for poultry are empty on arrival at the farm; restricting individuals and traders from entering the poultry houses where direct contact with poultry is possible; separating the farm into a clean and dirty area; preventing materials from being shared with other farms; implementing strict disinfection measures for the introduction of materials. When looking at internal biosecurity, disease management, referring to vaccination records, health monitoring, removal of dead birds, and stocking density, recorded high scores, while the category of cleaning and disinfection requires improvement. Practices that can be refined are: strictly dividing the clean and the dirty area of the house's hygiene lock; setting a hand washing facility in the hygiene lock; frequently cleaning the feed silo on the inside.

A pattern of improved biosecurity is observed in broiler farms visited at least three times. It is more obvious in external scores (they rose from about 67 to 82), while internal scores only indicate a minor increase (they increased from approximately 78 to 82) over a longer period. The results of the recommendations distribution indicate that farmers and workers might have not strictly followed the advice about internal biosecurity suggested by PVPs because C&D was one of the most frequently recommended categories.

Combining the distribution of recommendation categories (Fig. 5) and scores per category over years (Fig. 2), we found that the most common recommendations for broiler farms match those categories with noticeably lower scores. Specially, C&D (scores from 60 to 70) show this match. However, this finding does not apply to some other frequently recommended categories. For instance, infrastructure and biological vectors is the second most common category of recommendations for broiler farms, while it scores were relatively higher than most other categories (scores always higher than 80). A similar situation is observed for entrance of visitors and farm works, whose scores were above 75 from 2019 to 2021 and above 80 from 2022 to 2023 but yearly distribution of the recommendation category were around 20% from 2019 to 2022. This suggests that PVPs perceive risk differently, which means they might prioritise recommendations based on their cognition and experience instead of numerical scores. Further training for PVPs in this area could address this difference.

Analysing the changing patterns of categories with a high frequency of recommendations, some experienced a rise in scores. For example, the scores for infrastructure and biological vectors for broiler farms increased from around 85 to 95 from 2021 to 2022, with the proportion of recommendations reaching 40.6% in 2021. However, this trend is not observed in most categories with frequent recommendations. Many of them did not show improvement, and some even experienced a decrease in scores. For instance, removal of manure and dead animals saw a decline in scores from 2020 to 2021, while the proportion of recommendations achieved 20% in 2020. Another example is C&D, which consistently had high proportions of recommendations, but its scores remained flat from 2019 to 2023. Its implementation can be checked on-farm with microbiological swabs and basic checks of disinfectant use, and these checks allow appraisal of return on investment through before–after changes in key parameters such as meat yield, mortality and antimicrobial use. Once again, this suggests that farmers might not strictly follow the advice given by PVPs or are encountering difficulties when implementing the recommended practices. Further investigation is needed to understand how to enhance their motivation or support them more effectively in adopting these biosecurity measures.

The same questionnaire was previously utilized by Gelaude et al. [12] in a pilot study conducted on 15 broiler farms in Belgium from 2012 to 2013, as well as by Van Limbergen et al. [30], who applied it to 400 broiler farms across five European countries (Belgium, Finland, Greece, Poland, and Spain) in 2016. Gelaude et al. [12] reported a mean external biosecurity score of 64 and a mean internal score of 73, while Van Limbergen et al. [30] found slightly higher averages, with external and internal biosecurity scores of 68.4 and 76.6, respectively. In contrast, from 2019 to 2021, the external biosecurity scores in selected Irish farms (63.0, 65.5, and 64.5) were close to those observed in Belgium from 2012 to 2013 but fell slightly below the averages found in the five European countries in 2016. During the same period, internal biosecurity scores in Ireland (72.9, 75.4, and 76.0) were also marginally lower than results in five European countries. However, in 2022 and 2023, external biosecurity scores in the Irish farms (79.5 and 78.7) exceeded both prior studies, while internal biosecurity scores (79.2 and 79.9) also surpassed those found in the five European countries.

Several factors may explain these differences. Firstly, Gelaude et al. [12] only examined 15 farms, which may have limited the coverage of their results when compared to the broader scope of subsequent studies. Secondly, the variation in countries involved across these studies likely reflects differing national policies, regulations and risks in terms of animal health and farm management. The marked improvement in scores, in our population, after 2021 may also be attributed to increased biosecurity awareness following recent outbreaks of HPAI in Ireland [15, 22]. Notably, the gaps between internal biosecurity scores were smaller than those between external biosecurity scores, and internal scores were consistently higher across most countries. This pattern has also been observed in countries from other regions, such as Ethiopia and the Philippines [27, 32], reflecting a common emphasis on internal biosecurity. A possible reason for this emphasis is the positive impact of strong internal biosecurity measures on farm performance. Castro Burbarelli et al. [6] suggested that farms following a proposed cleaning and disinfection program showed better productive performance compared to those that did not. Furthermore, a subsequent study by Castro Burbarelli et al. [7] found that although a more detailed cleaning and disinfection protocol incurred higher costs, it achieved a similar gross margin to a simplified protocol. Therefore, a detailed cleaning and disinfection programme had economic viability. Meanwhile, it can provide better health conditions for broiler chickens, reducing the possibility of loss due to diseases. This benefit might be an important incentive for farmers to implement a higher standard of cleaning and disinfection in farms.

Several limitations of this study should be acknowledged. First, the assessments were conducted by different PVPs, introducing variability in scoring due to differences in interpretation and implementation of the BioCheck.UGent tool. Although PVPs received specific training, individual assessment approaches could affect consistency. Second, the data collection relied partially on self-reported information from farmers, which could potentially introduce bias, especially for procedures that are not observed in the day of visits (for example cleaning and disinfection). Third, this study relies on the scoring system generated by Biocheck.Ugent questionnaire, and specific practices were detected only when their corresponding categories scored poorly. Consequently, some suboptimal practices within well-scoring categories may not be captured. Fourth, though participation in later years increased to almost complete coverage, it was voluntary in the first year. Therefore, participating farms were probably more engaged with biosecurity, which may have raised baseline scores and reduced the likelihood of observing large improvements in the early years. In addition, farm types are also vital for biosecurity practices, but in this study, no related data were collected, though different types exist such as intensive, free range, and organic farming, limiting the analysis of how different production systems impact biosecurity compliance. Furthermore, the original questionnaire was retained throughout 2019–2023 to preserve longitudinal comparability. However, as refined versions have been released, some questions may no longer fully reflect current biosecurity practices.

## Conclusion

In conclusion, there is room for improvement of external biosecurity for Irish broiler farms. At the same time, internal biosecurity displays a relatively higher level of compliance for what can be measured via a survey, but can still be perfected, especially for C&D. The level of compliance with recommendations suggests that further supports are needed to enable better compliance from farmers. Presenting the cost–benefit of implementing different biosecurity measures to farmers so that information can help their decision-making process for better returns on their investments might be one of those supports needed. The data obtained through this study will be used for those cost–benefit estimations. Specifically, benefits will be estimated by changes in outcomes after implementing better biosecurity practices. Costs will be compiled from recorded inputs required to implement the measures, such as detergents, energy, equipment and labour fees.

## Data Availability

Access to the data is available on a reasonable request.

## Abbreviations

AHI: Animal Health Ireland
AMR: Antimicrobial Resistance
AMU: Antimicrobial Usage
C&D: Cleaning and Disinfection
HPAI: Highly Pathogenic Avian Influenza
PVPs: Private Veterinary Practitioners
TASAH: Targeted Advisory Service on Animal Health
